# Cavity-agnostic acoustofluidic manipulations enabled by guided flexural waves on a membrane acoustic waveguide actuator

**DOI:** 10.1038/s41378-023-00643-8

**Published:** 2024-03-08

**Authors:** Philippe Vachon, Srinivas Merugu, Jaibir Sharma, Amit Lal, Eldwin J. Ng, Yul Koh, Joshua E.-Y. Lee, Chengkuo Lee

**Affiliations:** 1 Department of Electrical and Computer Engineering, National University of Singapore, Singapore, Singapore; 2 Institute of Microelectronics, Agency for Science, Technology and Research (A*STAR), Singapore, Singapore; 3 SonicMEMS Laboratory, School of Electrical and Computer Engineering, Cornell University, Ithaca, NY USA; 4 School of Electrical and Data Engineering, University of Technology Sydney, Ultimo, NSW Australia

**Keywords:** Electrical and electronic engineering, Microfluidics

## Abstract

This article presents an in-depth exploration of the acoustofluidic capabilities of guided flexural waves (GFWs) generated by a membrane acoustic waveguide actuator (MAWA). By harnessing the potential of GFWs, cavity-agnostic advanced particle manipulation functions are achieved, unlocking new avenues for microfluidic systems and lab-on-a-chip development. The localized acoustofluidic effects of GFWs arising from the evanescent nature of the acoustic fields they induce inside a liquid medium are numerically investigated to highlight their unique and promising characteristics. Unlike traditional acoustofluidic technologies, the GFWs propagating on the MAWA’s membrane waveguide allow for cavity-agnostic particle manipulation, irrespective of the resonant properties of the fluidic chamber. Moreover, the acoustofluidic functions enabled by the device depend on the flexural mode populating the active region of the membrane waveguide. Experimental demonstrations using two types of particles include in-sessile-droplet particle transport, mixing, and spatial separation based on particle diameter, along with streaming-induced counter-flow virtual channel generation in microfluidic PDMS channels. These experiments emphasize the versatility and potential applications of the MAWA as a microfluidic platform targeted at lab-on-a-chip development and showcase the MAWA’s compatibility with existing microfluidic systems.

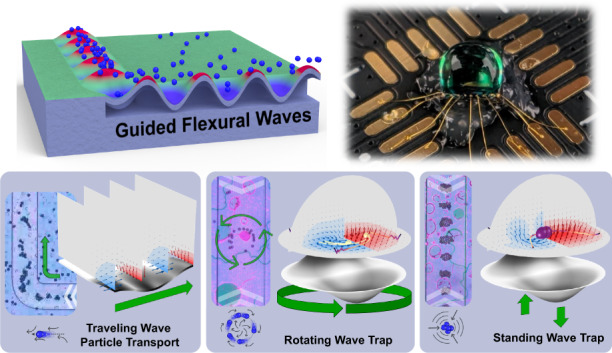

## Introduction

Acoustofluidics is a rapidly developing field that leverages the synergy between acoustics and fluid dynamics to manipulate fluids and particles at micro and nanoscales. The various effects generated through this approach have led to the development of acoustofluidic devices targeting critical biomedical applications like tissue engineering^1^, nanoparticle synthesis^2^, intracellular delivery^3–5^, spheroid formation^6^, whole blood components separation^7^, micromixing^8^, and organism^9–12^ and single-cell manipulation^13,14^. One may refer to Rufo et al.^15^ for a comprehensive review on acoustofluidics for biomedical applications and Friend & Yeo^16^ for a thorough introduction to acoustic microfluidics.

Two forces are predominantly being harnessed for acoustofluidic actuation, the acoustic radiation force (ARF) and Stokes’ Drag force, here designated as the acoustic streaming force (ASF)^17,18^. The ARF typically arises inside closed systems like channels and cavities by leveraging the resonant quality of the features in place. It represents the energy transferred from the traveling sound waves to a particle and has traditionally been the dominant force at play for particle manipulation, separation, and trapping. Acoustic streaming, on the other hand, arises from the nonlinear interaction between sound waves and fluid, leading to the generation of fluid vortices and flows. The ASF has long been considered an undesirable side-effect of ultrasonic actuation in several acoustofluidic systems and is often seen as acting against the ARF, reducing the precision and focus of the targeted acoustofluidic effects.

Typical acoustofluidic systems leveraging the ARF place the utmost importance on the geometry of the fluidic chamber or cavity. The position of the walls governs the propagation of the sound waves and the distribution of the acoustic field, essential for creating tools like acoustic tweezers^14,19,20^. Numerous acoustofluidic devices are hence designed for resonant standing wave generation. The half-wavelength resonator^21,22^ is a typical type of acoustofluidic system that relies on matching the frequency-derived acoustic half-wavelength with one of the cavity’s dimensions. These systems are often excited via bulk acoustic wave (BAW) and have proven to efficiently leverage the ARF to separate particles, platelets, and cells inside a free flow channel^23^ or focus metal and polystyrene particles inside a glass capillary^24^. The physics behind the half-wavelength resonators can also be applied to standing wave patterns acoustically excited via surface acoustic wave (SAW) inside a microfluidic channel. This type of actuation causes the position and the alignment of polystyrene particles to shift along a 2D plane within the channel^25^ following the established diffraction pattern^26–28^. Furthermore, by multiplying the number of sources generating SAW, a field of standing surface acoustic waves (SSAWs) can also be leveraged to form configurable acoustic lattices capable of advanced and selective spatial control of cells, particles and clusters^29^. It is also possible to couple surface waves to a glass capillary and generate a tridimensional standing wave pattern controlling the position of the particles inside the tube through the ARF^30^.

Although ARF-powered resonant devices have been prevalent, novel and innovative actuation technologies have successfully leveraged the ASF or both forces simultaneously to achieve acoustofluidic functions. By varying the height of a microfluidic channel, it is possible to adjust the intensity of the ASF while keeping the ARF stable. This cavity-based approach leveraging both forces enables size-dependent particle separation, alignment, and enrichment and has been investigated inside a microchannel excited by traveling SAW-driven diffractive acoustic fields^27,31^ and demonstrated inside a lateral flow microchannel featuring a lanceolate GHz bulk acoustic resonator^32^.

SAWs are known for *leaking*^33^ radiative energy into the surrounding fluid, while simultaneously inducing strong localized streaming phenomena. By adding a small volume of particle-laden liquid in a microwell or as a sessile droplet on top of the path of the propagating SAW, different mixing, aggregation, and particle clustering can occur^34–40^. Furthermore, the intensity of the streaming can be such that a small droplet can be displaced using SAWs^41–43^.

In such systems, however, the energy emitted by the transducers radiates through the fluidic medium, and the acoustic fields generated are contained by the boundaries of the cavity or the droplet. These fields often feature a *resonant* or *persistent* behavior as they interact with channel walls and other boundaries of the fluidic domain to create interference and diffractive acoustic fields. On the other hand, *evanescent* acoustofluidic fields have been reported in a small number of systems and present a promising direction for novel cavity-agnostic acoustofluidic mechanisms. These fields have the advantage of being highly localized due to the nature of the acousto-mechanical transducer employed and allow for the trapping and transport of particles when in proximity to the acoustic source. Vibrating structures actuated via BAW, such as resonating micropillars or sharp edges, have also revealed themselves as effective tools for particle trapping^44,45^, transport along complex trajectories^46^, and in-channel pumping^47^ and mixing^48–51^. Similarly, holographic cell patterning has also been demonstrated based on a 3D-printed phase-encoded plate mounted on a single transducer inducing localized acoustic streaming at the pressure nodes^52^.

In flexural-plate-based systems, the literature reports the use of piezoelectric transducers to generate interfering flexural waves, causing microparticles and cells to aggregate inside a multi-well plate^53^ and sessile droplets^54^. To enhance the acoustofluidic effects of traveling flexural waves, Liu et al.^55^ formed dimples at the interference focal points to amplify the displacement of the substrate, inducing particle trapping at those locations.

The first report of flexural waves being investigated in acoustofluidics^56^ is the fruit of Richard White’s group more than three decades ago. Their principal findings detail that the acoustic fields generated by flexural waves are evanescent in the normal direction from the plane of propagation (substrate) and that the amplitude of the propagating flexural wave decays much more slowly than that of radiating leaky waves in similar conditions. Hence, flexural waves can be harnessed to generate localized distributed pumping to transport microparticles^57–64^ and mix fluids^65^. They also reported several sensing and multi-sensing^66,67^ experiments for various parameters such as chemical vapor^68–70^, fluid density^71^, fluid viscosity^72,73^, cell growth^74^, and bacterial concentration^75^. However, the fabrication technology employed at the time required back-side etching for the membrane formation, which limits the designs to very large membranes and decreases the structural integrity of the chip.

**Fig. 1:**
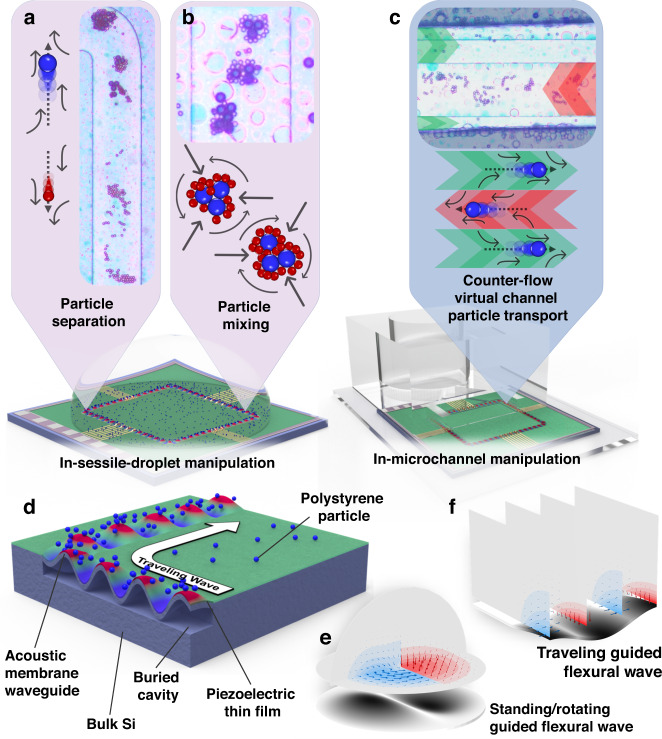
Advanced acoustofluidic manipulation on the MAWA platform. **a** In-sessile-droplet size-based particle separation. **b** In-sessile-droplet particle mixing. **c** In-microchannel counter-flow virtual channel generation for particle transport. **d** Schematic cross-section of the MAWA with traveling GFW causing blue particles to align and travel in the wave propagation direction (white arrow). **e** Simulated fields of the localized acoustofluidic forces for standing/rotating GFW in an open hemispherical fluidic domain. **f** Simulated fields of the localized acoustofluidic forces for a rectilinearly traveling GFW inside a microfluidic channel

Recently, novel acoustofluidic effects induced by traveling guided flexural waves (GFWs) have been demonstrated by Vachon et al.^76^ based on a lithographically-defined piezoelectric suspended membrane acoustic waveguide actuator (MAWA), Fig. 1d. The complex and detailed structures realized by the piezoelectric Silicon-On-Nothing (pSON)^77^ process allows for the MAWA to distinguish itself from the flexural plate wave transducers reported by White’s group by leveraging the waveguiding property of a photolithographically-defined membrane^78^ and further develop the capabilities of localized GFWs in acoustofluidics.

In the previous paper^76^, the mechanical properties of this waveguide-transducer pair were investigated to highlight the emergence of different GFW vibration modes populating the membrane waveguide of the MAWA. The in-sessile-droplet acoustofluidic investigation experimentally identified three primary modes and their respective acoustofluidic effect: (1) unidirectional traveling waves generate localized evanescent streaming capable of aligning and transporting microparticles, (2) counter-propagating waves interfering can lead into the formation of standing flexural waves capable of trapping particles into static clusters, (3) the latter interfering counter-propagating waves can also form rotating flexural waves capable of assembling particles into rotating ring traps.

Yet, as presented by Vachon et al.^76^, discerning the specific contributions of the ARF and the ASF to the acoustofluidic effects induced by GFWs proved challenging through experiments alone, prompting the need for detailed acoustofluidic simulations to highlight the distinction between these forces.

Hence, to build upon the previous work, this article presents essential acoustofluidic simulation results based on the abovementioned three primary vibration modes to elevate the understanding of the MAWA’s operation and be used as building blocks to support the experimental results. By doing so, one can understand and link a typical mechanical displacement on the flexural membrane to an expected acoustofluidic response. Furthermore, novel acoustofluidic particle manipulation functions are introduced through experiments targeting particle separation (Fig. 1a), particle mixing (Fig. 1b), and counter-flow virtual channel transport (Fig. 1c) to showcase the capability of the MAWA to control particles of different sizes simultaneously and highlight the localized and cavity-agnostic properties of the acoustic actuation.

The MAWA presents itself as a polyvalent solution for the development of multipurpose acoustofluidic platforms as the different modes of operation and their respective acoustofluidic effects can independently or simultaneously be combined to enable complex and dynamic particle control experiments inside any fluidic domain, such as sessile droplets and microfluidic channels. In this research article, the status of the MAWA as an essential cavity-agnostic technology for a consolidated microfluidic platform is further established.

The first part of this paper provides a visual and numerical representation of the inner mechanisms of three primary modes of GFWs. In the first model, a GFW rectilinearly traveling on a thin solid membrane is presented, showcasing the behavior of the ARF and ASF fields generated by the wave propagation inside a microchannel, Fig. 1f. In the second model, a standing flexural wave bounded to a circular membrane is featured, demonstrating the dominant ARF field and the weaker ASF field. In the third one, the standing wave model becomes a rotating (circularly traveling) flexural wave to showcase the dominant vortical streaming induced by this type of traveling wave, Fig. 1e.

The second part of the paper provides a series of acoustofluidic experiments showcasing advanced particle manipulation functions performed by the MAWA by leveraging the primary vibration modes explored numerically. The first experiment features in-sessile-droplet particle mixing and clustering (Fig. 1b) on the membrane waveguide and leverage the standing and rotating primary modes. The second experiment targets particle separation based on size (Fig. 1a) by harnessing the transport function of the primary traveling wave mode and the trapping capability of the latter two primary modes of the MAWA. The third experiment features a controllable virtual counter-flow channel arising from the localized evanescent distributed pumping generated by traveling GFWs (Fig. 1c). It highlights the compatibility of MAWA-based chips with standard PDMS microfluidic systems as well as the possibility for localized and controllable virtual microfluidic transport channels in potential biomedical applications such as particle washing and enrichment. Together, these advanced acoustofluidic experiments convincingly portray the range of cavity-agnostic particle manipulation abilities that the MAWA possesses.

### Description of the MAWA

The MAWA comprises two main parts: (1) an acoustic membrane waveguide to guide and confine the different traveling and standing modes of flexural waves and (2) interdigitated transducers (IDTs) formed by carefully spaced electrodes on top of a piezoelectric thin-film.

**Fig. 2:**
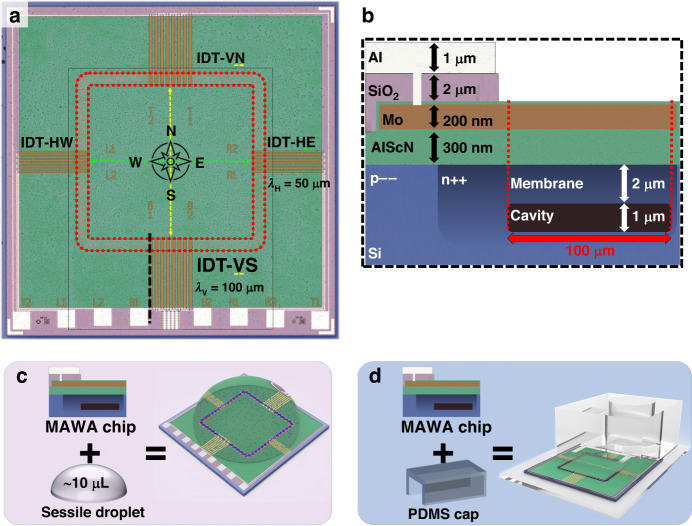
MAWA device layout and fabrication. **a** Top view of the MAWA device from an optical microscope. The red dashed lines indicate the boundaries of the membrane waveguide. The IDTs are identified as IDT- followed by the first letter indicating their shared axis and the second letter for the cardinal direction. The vertical black dashed line over IDT-VS represents the region where the schematic cross-section (**b**) is taken. **b** Schematic cross-section of the device with annotation of the layers’ material and targeted thickness, the doped region, the buried cavity, and the membrane waveguide. The SiO_2_ layer isolates the Mo electrodes of the IDTs from the Al layer of the bond pads and routing. **c** MAWA configuration for in-sessile-droplet acoustofluidic particle manipulation. **d** MAWA configuration for in-microchannel acoustofluidic particle manipulation

The piezoelectric Silicon-On-Nothing (pSON)^77^ process is used to fabricate the thin suspended membrane forming the waveguide. This process requires a high-temperature annealing step to induce silicon migration inside the patterned substrate, effectively sealing off a seamless vacuum cavity buried inside the bulk silicon. The thin Si membrane obtained by this step is then covered with a thin piezoelectric film. Upon actuation, the piezoelectric membrane and the interdigitated electrodes patterned on top generate a flexural mechanical deformation giving rise to traveling antisymmetric A0 flexural waves propagating on the membrane (Fig. 1a, d). As the membrane primarily acts as a waveguide for the waves by confining, guiding, and supporting the waves, the term guided flexural waves (GFWs) was coined to reflect this fundamental property. Due to the thinness of the membrane, GFWs travel on the latter at phase velocities slower than the speed of sound in water, preventing the wave energy from radiating inside the bulk fluid in contact with the membrane. Instead, the mechanical energy transferred is concentrated inside the first microns of the fluid, resulting in the generation of evanescent fields and confined cavity-agnostic acoustofluidic effects. As such, acoustofluidic manipulations performed using GFW can be realized indiscriminately inside a sessile droplet (Fig. 2c) or a microfluidic channel (Fig. 2d).

There are four IDTs on the MAWA device, each positioned in the middle of the four segments of the rectangular closed-loop membrane waveguide (see red dashed line in Fig. 2). The North (top) and South (bottom) IDTs, positioned to be vertically opposed on the North-South axis, have a period of ${\lambda }_{{\rm{V}}}$ of 100 μm and are grouped under the label of IDT-V (IDT-VN, IDT-VS). The East (right) and West (left) IDTs, positioned to be horizontally opposed on the East-West axis, have a period ${\lambda }_{{\rm{H}}}$ of 50 μm and fall under the label of IDT-H (IDT-HE, IDT-HW), Fig. 2a. The different periods of the IDTs and their paired position on opposite segments allow for the device to function inside a broader range of frequency and facilitate the simultaneous actuation of IDTs, increasing the selectivity and accuracy of the particle manipulation functions performed.

### MAWA chip fabrication

The MAWA chip featured in this work is a 3 mm × 3 mm microfabricated silicon chip bearing a 2 µm-thick monocrystalline silicon membrane forming a rectangular closed loop with rounded corners, Fig. 2a. The membrane itself, also referred to as silicon-on-nothing, is formed by seamlessly sealing off the buried cavity through silicon migration^79,80^, Fig. 2b. As such, the membrane can be photolithographically defined, resulting in far greater precision and flexibility in the definition of its shape than what conventional back-side etching offers.

On top of the silicon membrane is a 0.3 µm-thick piezoelectric layer of scandium-doped aluminum nitride (Al_0.85_Sc_0.15_N). Molybdenum (Mo) electrodes are patterned on the AlScN layer to define interdigitated transducers, essential for actuating the underlying suspended silicon membrane and thereby generating GFWs. A 2-µm silicon oxide (SiO2) layer is deposited and patterned on top of the electrode layer to act as a spacing layer between the bottom Mo electrodes and the top electrodes made from 1 µm aluminum (Al). The different fabrication layers are illustrated in Fig. 2b.

### MAWA primary vibration modes

The membrane waveguide of the MAWA supports various vibration modes which can be deconstructed into three primary localized modes for acoustofluidic manipulations, as identified by Vachon et al.^76^.

**Fig. 3:**
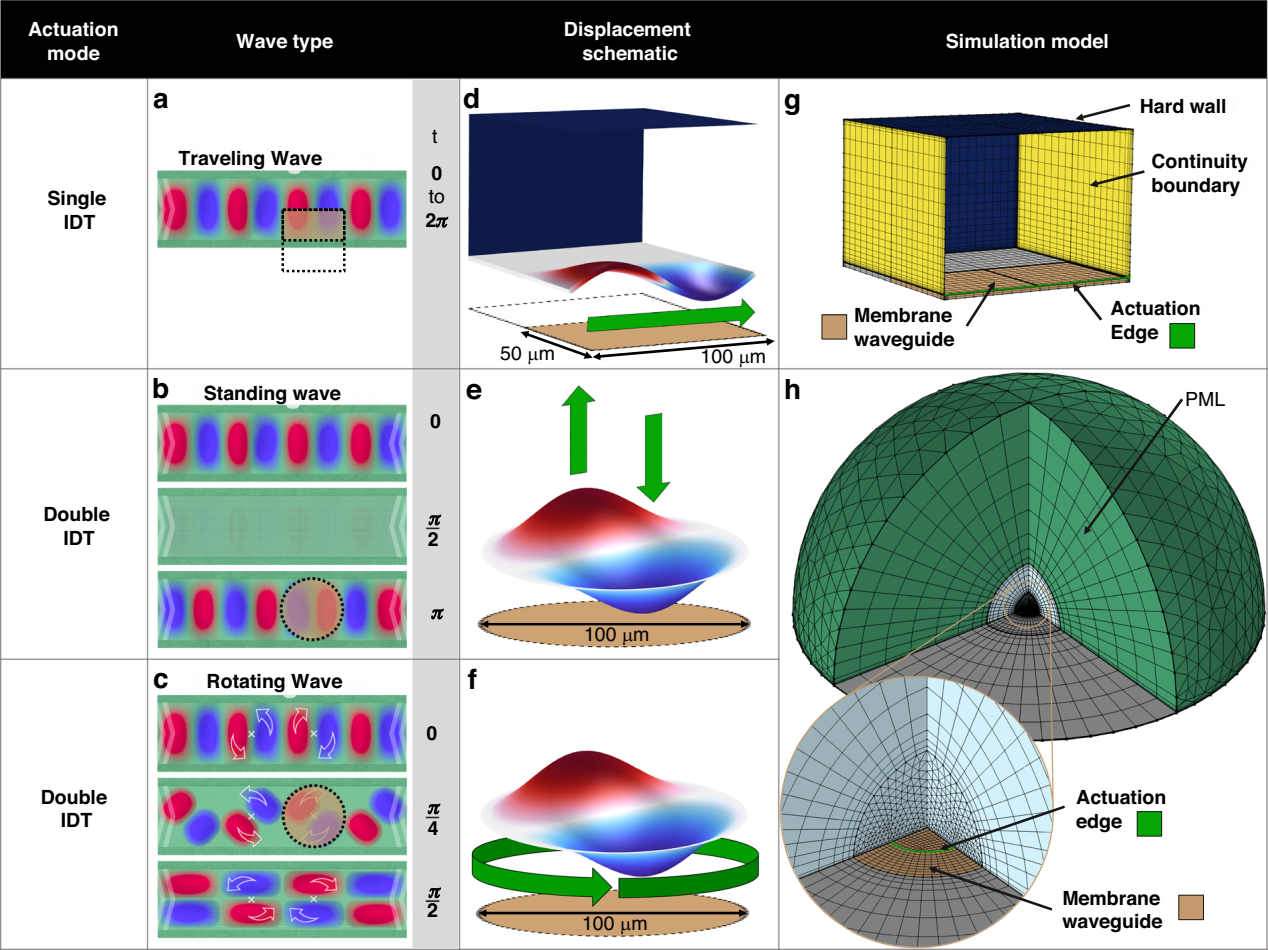
Numerical localized models of the three primary wave modes on the membrane waveguide. **a** Top view schematic of a traveling wave. The traveling wave arises under single IDT actuation, as illustrated by the single white chevron arrow on the left side, indicating the direction of propagation. **b** Top view schematics of a standing wave. A standing wave is generated under double IDT actuation, as illustrated by the white chevron arrows which indicate the traveling direction of counter-propagating waves coming from each side. **c** Top view schematics of an array of rotating waves. Rotating waves are generated under double IDT actuation (white chevron arrows) as off-axis counter-propagating waves interfere. The time *t* indicated on the right-hand side of the schematics in (**a**, **b**, **c**) is the time during a period of actuation. The sand-colored sections bounded by black dashed lines in (**a**, **b**, **c**) indicate the footprint of the numerical model in (**d**, **g**), and the footprint of only the flexural membrane waveguide of the numerical model in (**e**, **f**, **h**). **d** Numerical model of a single wavelength of a traveling flexural wave on the MAWA. **e** Schematic of a (1,1) standing wave on a circular flexural membrane. **f** Schematic of a (1,1) rotating wave on a circular flexural membrane. **g** Meshed traveling wave model with color-coded boundary conditions. **h** Meshed numerical model for the standing and rotating wave. The displacement schematics in (**e**, **f**) occur onto the sand-colored section representing the membrane waveguide. A PML section is wrapped around the fluid domain to act as an infinite domain. The gray bottom boundaries in (**g**, **h**) are fixed

The first mode is a simple traveling guided flexural waves of the A0 mode,Fig. 3a, which originates from one of the IDTs on the device. When a single IDT is continuously actuated through a sinusoidal signal, the membrane segments of the device in Fig. 2a will predominantly be populated by traveling waves, while the segment directly opposite of the source IDT will see the counter-propagating waves interfere and cancel the traveling motion of the wave due to the symmetrical loop-shape of the device.

The second mode is a standing wave formed when two counter-propagating traveling waves interfere perfectly, Fig. 3b. This gives rise to a standing A0 mode, without the traveling component, forming a linear array of node and antinode along the longitudinal direction of the membrane waveguide. This mode can be easily excited when two opposite IDTs, such as the vertical pair IDT-V or the horizontal pair IDT-H are simultaneously actuated.

The third mode is a rotating wave, a variant of the standing wave that instead arises when *bouncing* traveling waves with lateral momentum do not perfectly interfere with one another. This gives rise to a linear array of rotating waves akin to a circular (*m* = 1, *n* = 1) mode, rotating in clockwise-counterclockwise alternation, Fig. 3c, where *m* is the number of nodal diameters and *n* is the number of nodal circles.

The second and third modes, the standing wave and the rotating wave, can both be excited by actuating two opposing IDTs. The differentiating factors are the actuation frequency and the phase-shift between the two source IDTs, combined with the geometry of the device. For fixed geometry, changing any of the two above-mentioned factors will affect the effective wavelength on the membrane. For a standing wave pattern to fit on the geometry, some modes will require the wave to bounce and travel off-axis, which may lead to the formation of rotating waves instead of purely standing waves.

### Results: numerical investigation of GWF acoustofluidic effects

This section presents three multiphysics models for simulating the acoustofluidic effects of the three primary vibration modes of the membrane waveguide, the traveling, standing, and rotating GFWs. These three models are designed as building blocks from which more complex MAWA systems can be designed and explained. As such, the simulation model only depicts the acoustofluidic field around a segment of the membrane waveguide where these primary modes occur. The simulation of a traveling wave take place over the length of a single wavelength (100 μm) bounded by continuity boundary conditions, mimicking an infinitely long membrane waveguide, Fig. 3d, g.

The simulations for standing and rotating waves are performed on the same hemispheric model with a circular flexural membrane bearing a (1,1) mode, Fig. 3e, f.

For each model, the acoustofluidic fields and forces were simulated through finite element analysis in COMSOL Multiphysics ® (v6.1, Stockholm, Sweden), and further details on the models’ parameters are available in the electronic Supplementary Material.

### Domain separation, physics interfaces, and multiphysics coupling

The models studied in this paper follow a similar construction regarding the domains and physics distribution. The models are separated into two parts, a fluid domain on top and a dual-layer solid domain representing the membrane underneath. The Solid Mechanics physics interface is used to model the flexural displacement of the actuated membrane in the solid domain. The bulk part of the solid is fully constrained via a Fixed Constraint domain condition (in gray, Fig. 3g, h), while the membrane segment (sand-colored, Fig. 3g, h) is driven by an edge Prescribed Displacement condition actuated at a frequency of 2.8782 MHz for traveling and rotating waves and 3.20 MHz for standing waves and a maximum displacement amplitude $A$ of 12 nm. In all cases, the membrane full-width or diameter is 100 μm with a flexural wave wavelength $\lambda$ of also 100 μm.

In the fluid domain, the continuity equation and the Navier-Stokes equation are solved using the perturbation method^81,82^. The Pressure Acoustics physics interface oversees solving the first-order expansion of these equations to determine the velocity and pressure perturbation fields, while the Laminar Flow interface solves their second-order expansion to obtain the velocity and pressure fields. The coupling between the solid mechanics and the fluid physics is done via the Acoustic-Structure Boundary multiphysics interface, which converts the membrane’s motion into an acoustic source for the first-order fields.

Similarly, the first and second-order fields are coupled via two Acoustic Streaming Coupling multiphysics interfaces, one covering the boundary contribution and another for the fluidic domain contribution. The latter two implementations of streaming multiphysics are derived from the work of Bach and Bruus^83^, and Jorgensen and Bruus^84^ and are readily available in COMSOL. All models were solved using a frequency domain study followed by a stationary study.

### Numerical model for traveling GFWs in acoustofluidics

The first model aims to provide insight into the localized acoustofluidic fields generated by the first primary vibration mode identified on the membrane waveguide of the MAWA, a linearly traveling GFW, Fig. 3a, d. The substrate bearing the traveling wave is encased by a shallow PDMS channel enabling the microchannel ensemble to work under different pressure flow conditions. The model features a simple rectangular channel with periodic continuity boundary conditions at both ends and hard wall boundaries on the sides, Fig. 3g. The flexural waves propagate in the x-direction following $A{{\rm{e}}}^{-i\,^{2\pi x}/_\lambda }$ and an xz symmetry plane cuts the model in half. The channel height is 70 μm, and its half-width, from the wall to the symmetry plane, is 100 μm. The fluid domain is solved under three different physics interfaces as a 0th-order Laminar Flow interface is added to solve the constant pressure-driven background flow, using the pressure variation $\Delta {\rm{p}}$ between both ends of the channel as a variable.

**Fig. 4:**
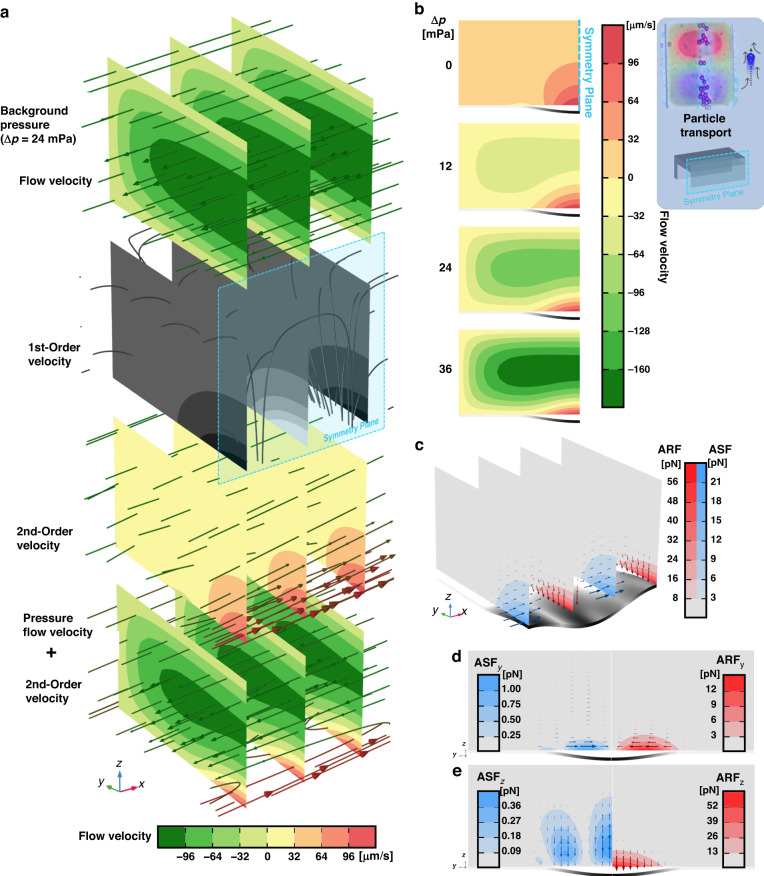
In-channel 3D simulation results of the acoustofluidic effects of localized linearly traveling GFWs inside a rectangular microfluidic channel. A longitudinal symmetry plane cuts (**a**, **b**, **c**) in half. The top right blue-colored inset shows the experimental phenomena expected from the model and the geometry of the model with the symmetry plane. **a** Simulation results displayed as stacked cross-sections of the background velocity fields, 1st and 2nd-order perturbation velocity fields. The last bottom stack shows the combined summation of the background and streaming (2nd-order) fields inside a rectangular channel under linearly traveling GFW actuation. The 1st-order velocity field is depicted in grayscale as it has zero time-average contribution in the system despite its magnitude greatly surpassing the 2nd-order field. **b** Transverse cross-section of the simulated combined background and streaming velocity fields displayed at different pressure variation $\Delta {\rm{p}}$ to show the development of the bi-directional flow shape. **c** Simulation results displayed as stacked cross-sections of the acoustofluidic forces generated by the traveling GFW inside the channel, with the ASF (blue) dominantly pushing in the direction of wave propagation (x-direction) and the ARF (red) pushing downwards (z-direction) toward the membrane waveguide. The forces are uniformly distributed in the x-direction. **d** Transverse cross-section of the y-component of the ASF (left) and ARF (right) showing both forces pushing toward the spinal ridgeline of the traveling GFW. **e** Transverse cross-section of the z-component of the ASF (left) and ARF (right) showing a dominant downward ARF_z_. In (**d**, **e**), both forces act on both sides of the symmetry but are selectively represented in half of the domain for easy comparison and interpretation

The results of the acoustofluidic simulations for the traveling flexural waves enclosed inside a PDMS microchannel are illustrated in Fig. 4. The different velocity fields of the 0th, 1st, and 2nd-order are shown in Fig. 4a. The 0th-order velocity field represents the fluid flow generated under a static pressure difference between both ends of the channel. Note that the 1st-order field is depicted in grayscale in Fig. 4a as it holds zero time-averaged contribution and is hence not involved in streaming generation. The 2nd-order velocity field, on the other hand, has a constant time-average contribution and is regarded as an important contributor to the streaming. The total velocity flow comprising both contributions from the 0th-order pressure flow and 2nd-order velocity flow are combined and summed together to show the formation of a localized counter-flow virtual channel powered by the traveling GFW-induced streaming flow coexisting with the traditional pressure flow ubiquitously used in microfluidics.

The amplitude of the 2nd-order streaming counter-flow is illustrated against four different background pressure flow induced by a pressure variation $\Delta {\rm{p}}$ ranging from 0 to 36 mPa, Fig. 4b. The simulation shows that the streaming flow is indeed evanescent in the normal direction away from the membrane and is further confined to a smaller region above the membrane when the pressure flow intensifies.

The two principal acoustofluidic forces, ASF and ARF, are illustrated in Fig. 4c–e. The tri-dimensional representations of the forces generated by traveling GFWs are shown in Fig. 4c. The ASF pushes particles in the direction of propagation of the wave (x-direction) and is more intense closer to the membrane waveguide as it is evanescent. Similarly, the ARF also decreases rapidly away from the membrane, but its principal component pushes particles downward in the z-direction. For a non-decaying traveling flexural wave, as it is the case in this model, the fields for both forces are uniform in x-direction.

The lateral contributions from the ASF and the ARF are shown in Fig. 4d, e for the y-components and z-components, respectively. When comparing both forces in the y-direction, it appears that the ASF and the ARF both contribute to the alignment of the particles along the central spinal ridgeline of the traveling wave, Fig. 4d. On the other hand, the ARF strongly dominates in the z-direction and pushes particle downward toward the membrane waveguide, Fig. 4e.

The simulation of the acoustofluidic forces generated by the first primary mode of the MAWA, a traveling GFW, reveals that particles are pushed in the wave direction of propagation while also being attracted to the maximum displacement line, formed at the spinal ridgeline of the traveling wave, along which they align.

### Numerical model for standing GFWs in acoustofluidics

As presented by Vachon et al.^76^, interfering GFWs will form different vibration modes over the membrane waveguide on which they propagate. The second primary mode identified arises when two waves traveling in opposite directions perfectly interfere and generate a standing wave on the membrane waveguide of the MAWA.

**Fig. 5:**
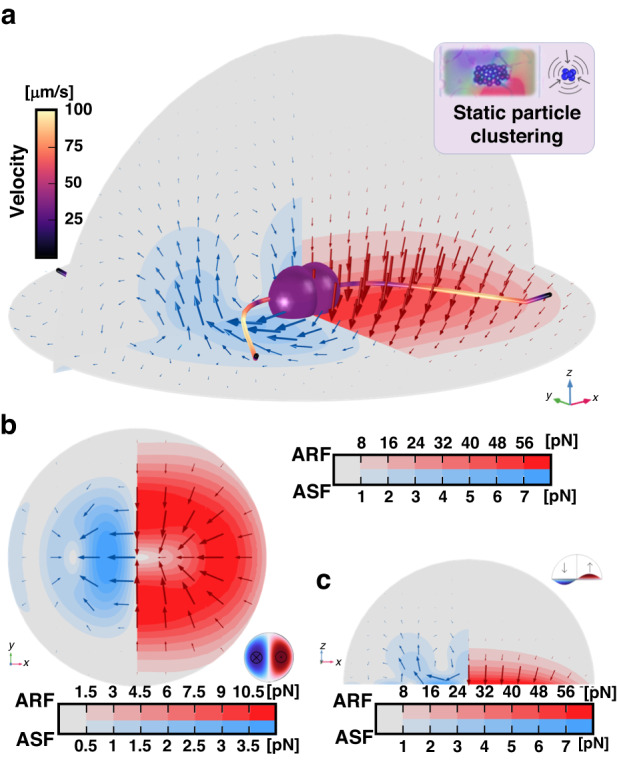
In-sessile-droplet 3D simulation results of the acoustofluidic effects of localized standing GFWs inside an open fluidic domain. The forces are presented selectively on their respective halves of the model to facilitate comparison and interpretation. It should be noted that the mirrored fields of both the ASF (left) and the ARF (right) exist on the opposite side of the symmetry cut-plane, although they are not displayed in the figure. The top right pink-colored inset shows the experimental phenomena expected from the model. **a** 3D simulation results showing the dominant ARF and the ASF under standing wave actuation. Particles are gathering at the center under the two acoustofluidic forcessw and their trajectories and velocity are traced. **b** xy-plane top view (z = 2 μm) of the simulated in-plane ASFxy and ARFxy showing the dominant ARF pointing toward the center node. The bottom right inset of (**b**) shows the (1,1) standing wave displacement field in the xy-plane. **c** xz-plane transverse view of the simulated in-plane ASFxz and ARFxz showing the dominant ARF pointing downward and toward the center node. The top right inset of (**c**) shows the (1,1) standing wave displacement field in the xz-plane

This model aims to provide insight into the localized acoustofluidic fields generated by a localized standing wave composed of two antinodes around a node. It features a hemispherical liquid domain on top of a circular bi-layer solid domain representing the bulk material and the suspended membrane, Fig. 3h. A displacement boundary condition is applied on a circular edge at $r=\lambda /4$ on the surface of the membrane waveguide to reproduce the (1,1) standing wave mode occurring from interfering traveling waves, see Fig. 3e and insets in Fig. 5b, c. Although the experimental equivalent of this model does not take place on a circular membrane, the circular domain facilitates the implementation of the physics and meshing while enabling the model to easily be transposed into and compared to the third primary mode, the rotating wave.

The acoustofluidic forces, the ARF and ASF, induced by the 1st and 2nd-order velocity fields and 1st-order pressure field were calculated to illustrate their general effect on 10 µm particles at 3.20 MHz.

Under periodic actuation, the oscillating mechanical displacement of the membrane forming a standing flexural wave gives rise to an intense ARF field pushing the particles with a positive acoustofluidic contrast factor (often the case for polystyrene particles) toward the central displacement node, forming an acoustofluidic trap. This force acts against the ASF, pointing toward the displacement anti-nodes. The simulation results of the field distribution of both the ARF (red) and the ASF (blue) are illustrated in Fig. 5. For 10 µm particles at 3.20 MHz, particles form a cluster at the node.

### Numerical model for rotating GFWs in acoustofluidics

**Fig. 6:**
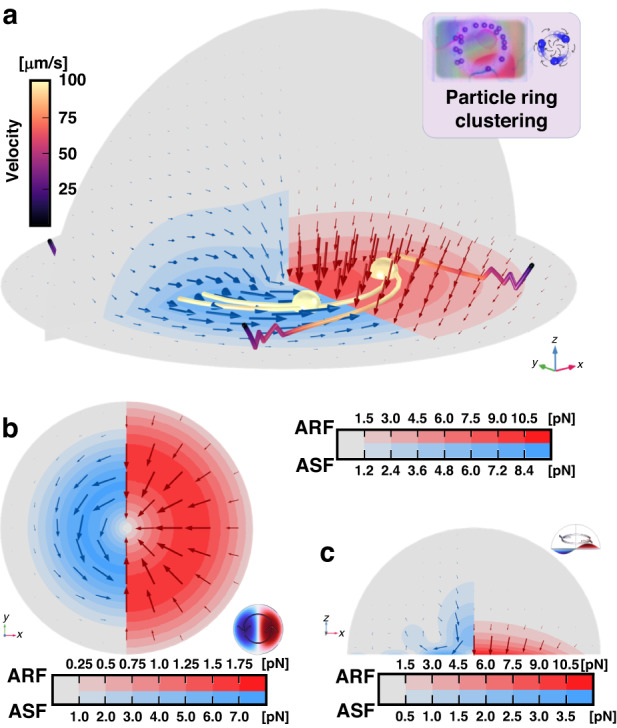
In-sessile-droplet 3D simulation results of the acoustofluidic effects of rotating GFWs inside an open fluidic domain. The forces are presented selectively on their respective halves of the model to facilitate comparison and interpretation. It should be noted that the mirrored fields of both the ASF (left) and the ARF (right) exist on the opposite side of the symmetry cut-plane, although they are not displayed in the figure. The top right pink-colored inset shows the experimental phenomena expected from the model. **a** 3D simulation results showing the dominant rotating ASF and the downward ARF under rotating wave actuation. Particles form a rotating ring under the two acoustofluidic forces and their trajectories and velocity are traced. **b** xy-plane top view (*z* = 2 μm) of the simulated in-plane ASFxy and ARFxy showing the dominant ASF rotating around the central node with the ARF pointing toward it. The bottom right inset of (**b**) shows the (1,1) rotating wave displacement field in the xy-plane. **c** xz-plane transverse view of the simulated in-plane ASFxz and ARFxz showing the dominant ASF pointing toward the displacement anti-node and the ARF pointing downward. The top right inset of (**c**) shows the (1,1) rotating wave displacement field in the xz-plane

In the second case of two waves traveling in opposite direction, the waves may not totally interfere due to the presence of a lateral momentum induced by the geometry of the device and a rotating wave may arise. Similarly, the model also takes place inside a hemispherical fluid domain on top of a bi-layer solid domain, Fig. 3h. The rotational actuation originates from a circular edge place at $r=\lambda /4$ featuring a periodically rotating displacement in the *z*-direction. Under periodic actuation, this displacement forms a rotating wave represented by a (1,1) circular mode with two anti-nodes traveling around a node in a rotational motion; see Fig. 3e and insets of Fig. 6b, c.

The acoustofluidic forces, the ARF and ASF, induced by the 1st and 2nd-order velocity fields and 1st-order pressure field were calculated to illustrate their general effect on the formation of rotating ring trap for 5 µm particles at 2.8782 MHz, Fig. 6.

The acoustofluidic solution of this model reveals strong streaming flow in the direction of the rotation, akin to what one may expect from a traveling wave bound to the smallest ring-shaped waveguide. The streaming can reach velocities in the hundredth of µm per second and drag particles around via the ASF (blue); see Fig. 6a, b. Apart from the main rotational streaming field, a perpendicular vortical streaming is also present at the anti-nodes, similar to the vortical streaming presented for the linearly traveling wave in Fig. 4. This streaming aligns the particles on the spinal ridgeline of the rotating wave, forming a rotating ring trap^76^. Once again, the main contribution of the ARF (red) is to push particles downward, preventing them from escaping the trap and keeping them in contact with the membrane, as the in-plane ARF (xy-plane) is too weak to compete with the ASF with the current parameters.

### Influence of the parameters on balance between the acoustofluidic forces

The standard expressions defining the ARF and the ASF used in this article are detailed in this section to highlight some of the shortcomings of the approach presented and provide insightful notes on the different parameters affecting the acoustofluidic effects from the MAWA.

1 $${\rm{ARF}}=-{\rm{\pi }}{a}^{3}\left[\frac{2{{\rm{\kappa }}}_{0}}{3}\mathrm{Re}\left({f}_{0}^{\star }{p}_{1}^{\star }\nabla {p}_{1}\right)-{{\rm{\rho }}}_{0}\mathrm{Re}\left({f}_{1}^{\star }{{\boldsymbol{v}}}_{1}^{\star }\cdot \nabla {{\boldsymbol{v}}}_{1}\right)\right]$$

Equation (1) is the acoustic radiation force on a micrometer-sized particle of radius $a$, compressibility ${{\rm{\kappa }}}_{{\rm{p}}}$ and density ${{\rm{\rho }}}_{\text{p}}$ floating inside a liquid of compressibility ${{\rm{\kappa }}}_{0}$ and density ${{\rm{\rho }}}_{0}$. The operator ^⋆^ denotes the complex conjugate of the variable, and the $\mathrm{Re}()$ operator preserves the real part. The perturbations from the incident acoustic field are given by the 1st-order pressure ${p}_{1}$ and the 1st-order velocity ${{\boldsymbol{v}}}_{1}$. The coefficients ${f}_{0}$ and ${f}_{1}$ are the monopole and dipole scattering coefficients, respectively^85,86^, see Eq. (2).

2 $${f}_{0}=1-\frac{{{\rm{\kappa }}}_{\text{p}}}{{{\rm{\kappa }}}_{0}},\,{f}_{1}=\frac{2\left({{\rm{\rho }}}_{\text{p}}-{{\rm{\rho }}}_{0}\right)}{2{{\rm{\rho }}}_{\text{p}}+{{\rm{\rho }}}_{0}}$$

Equation (3) represents Stokes’ law which provides the drag force, or ASF for the topic of this article, exerted on a spherical particle of radius $a$ moving at velocity ${{\boldsymbol{v}}}_{{\rm{p}}}$ in a fluid of viscosity ${\rm{\mu }}$ with a time-average 2nd-order velocity perturbation $\left\langle {{\boldsymbol{v}}}_{2}\right\rangle$.

3 $${\rm{ASF}}=6{\rm{\pi }}{\rm{\mu }}a\left(\left\langle {{\boldsymbol{v}}}_{2}\right\rangle -{{\boldsymbol{v}}}_{{\bf{p}}}\right)$$

The assumptions used to derive the expressions for the ARF and the ASF in Eqs. (1) and (3) consider a relatively uniform perturbation field around a single particle, which is not precisely the case for the evanescent acoustofluidic fields generated by GFWs. These fields extend straight up from the interface between the solid membrane and the fluid and can completely vanish at a distance comparable to a particle’s size, rendering the force expressions numerically inexact. However, the functional effects and directions of particle transport and trapping conveyed by the numerical results are still valid approximations of what one may expect from experimental results.

One experimental parameter that affects simulations and experiments is the driving frequency of the MAWA. At higher frequencies, the ARF is amplified, which affects the balance between the ASF and the ARF. This balance transition can be easily observed in trapping effects arising in modes like the rotating ring (Fig. 6) and static cluster trap (Fig. 5). At higher frequencies, the ASF lateral vortical streaming forming the ring trap may be surpassed by the ARF, which pushes the particles toward the displacement node and leads to the formation of a rotating cluster trap. However, the ASF may completely dominate over the ARF at lower frequencies. For standing waves, this can lead to particle cluster trapping happening at the displacement anti-node, as reported by previous works from Liu et al.^55^, Bachman et al.^87^, Vuillermet^88^, and Lei^89^.

A second experimental parameter that strongly affects the results is the size of the particles. As the ARF scales to the cube of the particle radius $a$, it can quickly dominate over the ASF for large particles, as experimentally shown later in this work. This is especially true with the short and exponentially decaying magnitude of the acoustofluidic effects coming from flexural waves. For submicron particles, the total ASF may be affected by the viscous boundary streaming, which is known to alter the trapping location of particles based on their size, as reported by Dorrestijn et al.^90^.

### Results: Experimental investigation of advanced acoustofluidic functions enabled by the MAWA

The acoustofluidic fields generated by the MAWA primarily depend on the membrane shape and the actuation mode. This dependence renders the MAWA-generated fields cavity-agnostic, unlike acoustic fields obtained through conventional acoustofluidic technologies based on BAW and SAW. These waves often rely on the shape of a cavity to establish the optimal acoustic fields. Furthermore, acoustic sources like SAWs are known to be strongly radiative, which directly affects the distribution of the acoustic fields as interference patterns arise inside a confined fluid domain, such as a microchannel or a small droplet. On the contrary, the evanescent and intrinsically localized acoustofluidic effects generated by the MAWA are decoupled from the shape of the fluidic chamber and can operate in any fluid domain above the membrane. This particular property conferred to MAWA-induced acoustofluidic effects makes the MAWA highly conducive to applications targeted at particle control. Hence, the MAWA can capture particles on top of the acoustic membrane waveguide and organize and transport them dynamically without the need for standard microfluidic tools like microchannels and benchtop pumps. To provide evidence for these claims, advanced particle manipulation experiments are presented in the following sections.

### Particle mixing experiment

**Fig. 7:**
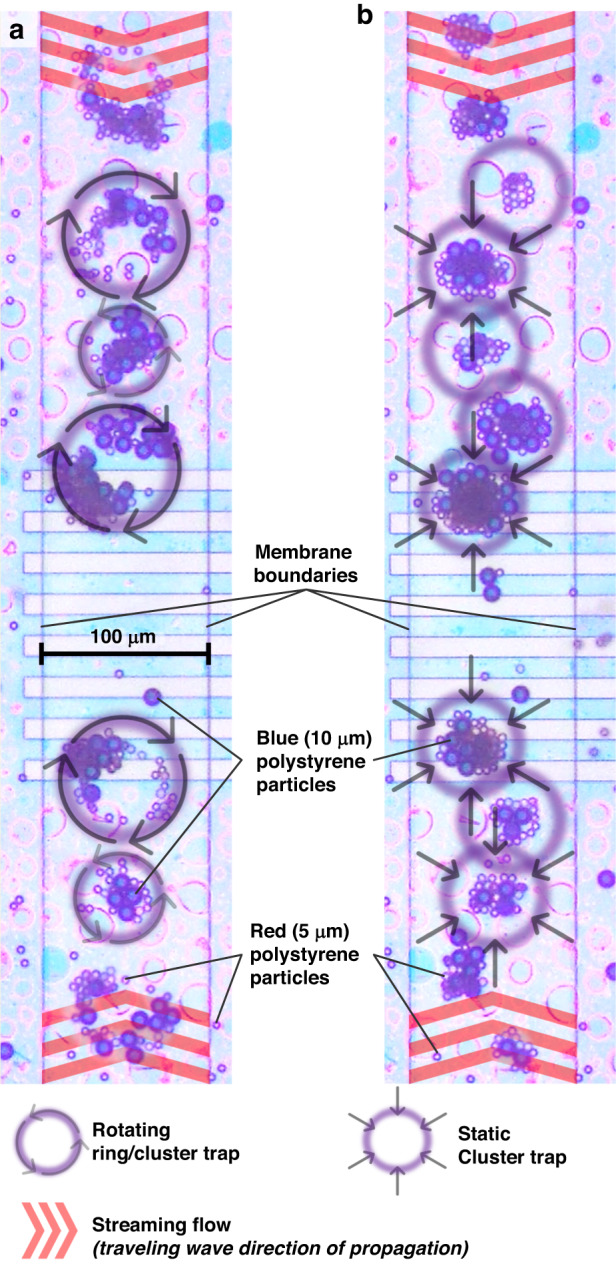
Experimental results of particle mixing and clustering by the MAWA inside a sessile droplet. **a** Optical microscope image of active mixing of 10 μm (blue) and 5 μm (red) particles on top of the membrane waveguide via localized vortical streaming from rotating traps at 2.85 MHz. **b** Optical microscope image of clustering of different particles on top of the membrane waveguide via standing-wave-induced trapping at 2.77 MHz. See supplementary movie S1

The first experiment involves two groups of polystyrene particles, 10 µm diameter blue particles and 5 µm diameter red particles, released inside a ~ 10 µL sessile droplet placed on top of the MAWA device. At the initial stage, the particles are randomly scattered on the device’s surface. Upon activating both IDT-Vs of the MAWA, traveling GFWs propagate on the membrane waveguide, and the induced streaming flow, as illustrated in Fig. 4, aligns and transports particles that had settled down on the membrane. The symmetric geometry of the membrane waveguide used causes the horizontal midsection of the device to act as a focal point where counter-propagating waves coming from IDT-VN and IDT-VS converge toward and interfere with one another, see Fig. 2 for the shape of the membrane and position of the different IDTs. Selected particles transported to this focal region via the localized ASF are then captured inside acoustic traps. These traps are formed by constructive interference between the waves coming from the opposite sides of the membrane waveguide. As Vachon et al.^76^ explained, different propagation and interference modes may arise on the MAWA, such as traveling, standing, and rotating waves. Combining the modes makes it possible to form traps with different cluster geometries, like rotating clusters and ring traps, which offer great potential for mixing particles of different types and diameters. This capability of the MAWA to gather and mix together particles of different sizes inside a sessile droplet is shown in Fig. 7.

Figure 7a shows blue and red particles captured and concentrated inside rotating traps. The particles were initially scattered inside the sessile droplet, but particles precipitating down onto the MAWA’s membrane surface were transported by the GFWs-induced localized streaming and concentrated at the focal region, as shown in Fig. 7a. As explained in the simulation section, rotating traps are formed when counter-propagating waves *bouncing* laterally on the membrane waveguide partially interfere and form an array of rotating waves. The rotating streaming arising around the traps actively mixes the particles and increases the contact between the different groups. The vortical traps in Fig. 7a were produced at a frequency of 2.85 MHz. However, by shifting the frequency to 2.77 MHz, the rotational components of the traveling wave vanish, and standing waves occur, leading to the clusters losing their rotational momentum and becoming static, Fig. 7b. The static clusters containing both red and blue particles now form a linear array of traps in the focal region of the membrane waveguide. A video of the in-sessile-droplet particle mixing and clustering experiment is available as the supplementary movie S1.

This localized mixing and clustering of particles inside a small fluid volume has several applications in biomedical research, such as analyte capture using functionalized microparticles and fast mixing in small volumes for agglutination assay^91^. Hence, the acoustofluidic capability of the MAWA places it as a suitable candidate for future lab-on-a-chip technologies.

### Particle separation experiment

As shown in Fig. 7, the primary modes of the MAWA can easily concentrate and mix different particles or analytes together. Nevertheless, by carefully enabling different modes simultaneously, it is also possible to spatially separate two groups of particles based on their size.

For this experiment, the two groups of particles, blue 10 µm and red 5 µm, were added to a sessile droplet on top of the MAWA device and mixed using the above-mentioned method. Then, the group of mixed particles was transported to the top segment of the device (North in Fig. 2) via GFW-induced streaming. The experiment utilizes three of the IDTs on the device, IDT-HE, IDT-HW and IDT-VN (Fig. 2). Once the particles were in place near IDT-VN, the two lateral IDT-Hs (E and W) (50 µm period) were actuated simultaneously at 9.8 MHz, 10 V_pk-pk_, to generate traveling GFWs propagating on the membrane toward the top of the device. The counter-propagating traveling waves originating from the two IDT-H sources constructively interfere on the membrane waveguide, forming a dominant standing wave. This standing wave pattern is the strongest on the North (top) membrane segment at the mid-distance mark between the two sources. Hence, the actuation of the two IDT-Hs causes an array of acoustic cluster traps to populate the top segment of the membrane. Shortly after, IDT-VN was independently actuated at 3.19 MHz, 10 V_pk-pk_, to act as a source of propagating GFWs.

On the membrane, the original standing mode sustained by the lateral IDT-HE and IDT-HW is upgraded into a mixed mode composed of standing waves gathering around the top segment of the close-loop membrane waveguide and traveling GFWs emanating from IDT-VN. As a result, particles caught onto the top segment of the membrane are subjected to both acoustic trapping (ARF), pinning them down, and acoustic streaming (ASF), transporting them away from IDT-VN. However, both these effects vary in intensity along the membrane waveguide due to the slowly decaying amplitude of the GWFs. Furthermore, the intensity of the two forces enabling the above-mentioned effects scales based on the particle properties. The ARF acts on the whole volume of a particle and scales to the cube of the radius, while the derivation for the ASF integrates along the area of the cross-section of the particle and scales linearly with the particle radius, see Eqs. (1, 3).

**Fig. 8:**
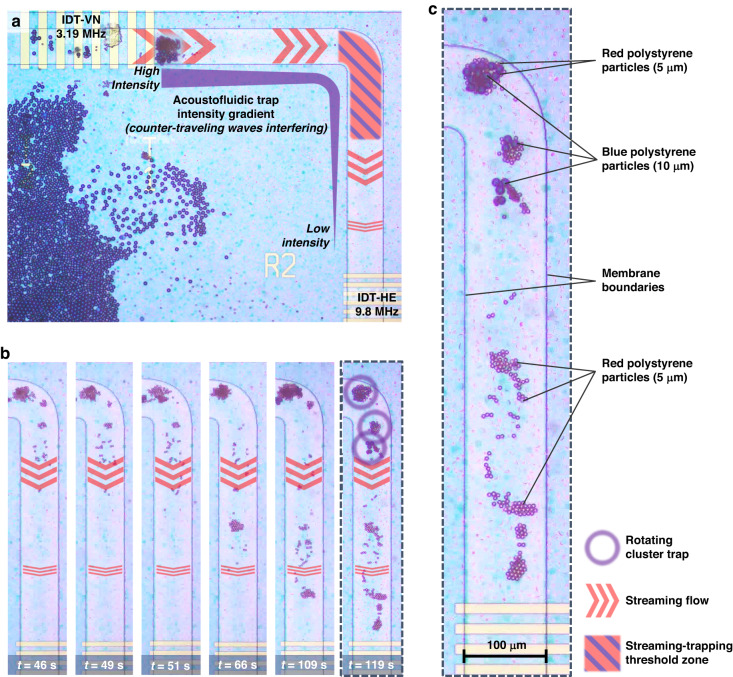
Experimental results of particle separation based on their diameter size by the MAWA inside a sessile droplet. **a** Optical microscope image of the active region of the membrane waveguide showing the initial cluster of particles to be separated. The annotations highlight the decaying streaming flow originating from IDT-VN and the acoustofluidic trap intensity gradient decreasing clockwise along the membrane waveguide. **b** Time-stamped sequence of optical microscope images showing the progressive formation of two spatially separated groups of particles. **c** Upsized copy of the last image in the sequence in (**b**) annotated to indicate the mixed group of 5 and 10 µm particles at the top and the bottom group solely composed of 5 µm particles

The variation in the intensity of both trapping and transportation effects depends on the particles’ position on the membrane waveguide and their radius. This dependence implies that specific behavior can be observed on different parts of the membrane from different groups of particles. Specifically, the experiments have shown that bigger particles are more likely to be trapped inside cluster traps generated by standing waves due to the characteristic cubic scaling of the ARF in relation to the particle radius. In comparison, smaller particles may evade the traps if the traveling-wave-induced ASF is large enough. This phenomenon is demonstrated in the diameter-based particle separation experiment seen in the supplementary movie S2 and explained in Fig. 8.

Figure 8a shows the entire active region of the particle separation experiment annotated with colored and shape-changing marks signaling the variation in amplitude of the forces in effect as a function of the position along the waveguide. The acoustic streaming (shrinking red chevron arrows) originating from IDT-VN slowly decays in intensity clockwise as it moves away from the source. Similarly, the acoustic traps dominate the top segment of the membrane waveguide and decay in the clockwise direction (thinning purple triangle). From this spatial dependence emerges a threshold zone inside which the dominant force changes from the ASF to the ARF, specifically for blue particles. The results reveal that by decreasing IDT-Hs voltage from 10 to 6.5 V_pk-pk_, large blue particles are left behind and stay trapped inside the ARF-dominant clusters while the smaller red particles, less susceptible to be driven by the now weakened ARF, follow the clockwise streaming and gather further away in a large group free of blue particles, The sequential frames illustrating sample’s time transition from one particle cluster to two distinct groups are shown in Fig. 8b and further magnified and annotated in Fig. 8c. A video of the in-sessile-droplet particle separation experiment is available as the supplementary movie S2.

Hence, the multi-modal capability of the MAWA allows for the spatial segregation of a mixed group of particles into different groups based on their size. Spatial partitioning of particles inside a sessile droplet is a novel approach for droplet-based manipulation and may offer significant improvements in biomedical applications such as cell sorting, cytometry, and immunoassay. By combining advanced localized particle control and particle separation, the MAWA emerges as a fully-fledged acoustofluidic solution for the development of lab-on-a-chip systems.

### Microfluidic channel counter-flow generation by traveling GFWs

The microfabrication technology used for the MAWA enables large-scale fabrication of the devices and ensures reproducibility and consistent performance from one device to another. Furthermore, the absence of back-ports during the fabrication process means that the chip’s structural integrity is preserved, and the MAWA can safely be integrated along standard microfluidic technologies such as PDMS microchannels. Although the MAWA can be used as a self-contained acoustofluidic platform, its integration with microchannels opens the door for high-throughput in-flow applications. Hence, in the next experiment, a wide microchannel is added on top of half of the device to showcase the formation of a localized counter-flow virtual channel inside the PDMS microchannel when the MAWA is actuated.

**Fig. 9:**
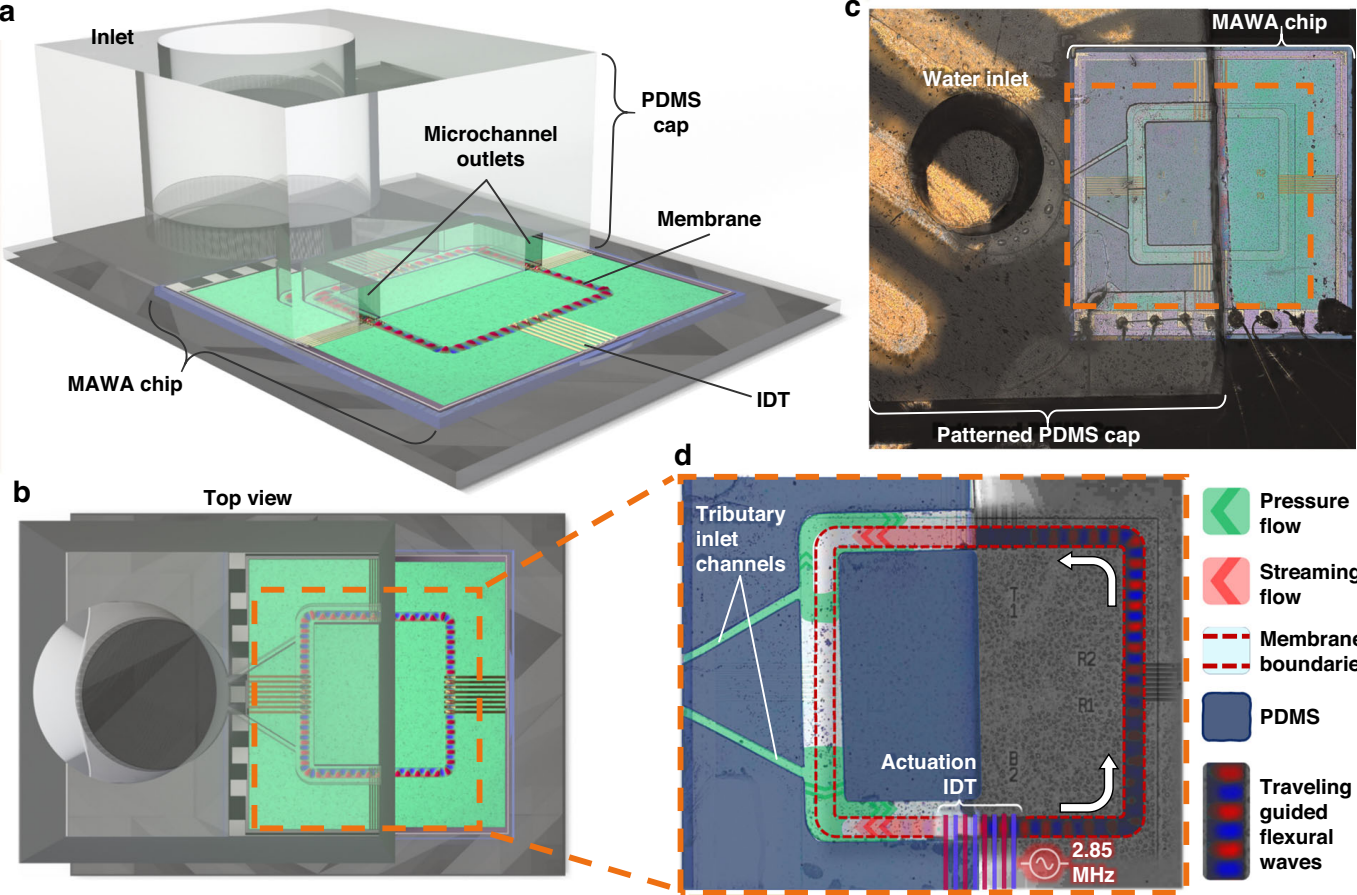
Configuration and functioning of the MAWA for in-channel microfluidic experiments. **a** Schematic of the MAWA chip outfitted with a patterned PDMS cap. The cap covers only the left half of the chip, while the right half is exposed. The microfluidic channels in the PDMS layer are aligned to the membrane waveguide on the chip. **b** Top view of the schematic featuring the top PDMS cap and underlying MAWA device. **c** Optical microscope image showing the patterned PDMS cap and the underlying MAWA chip before the experiment. In both (**b**, **c**), the orange dashed box identifies the region in (**d**). **d** Top view colored optical microscope image of the PDMS + MAWA device during the experiment. Particle-laden fluid is pumped from the left through the tributary inlets to reach the active membrane encased in the microfluidic channel. IDT-VS at the bottom of the membrane loop is actuated, resulting in a burst of traveling GFWs inducing a localized streaming counter-flow. The waves are imaged by LDV and superposed on the optical image for visualization purposes. The opposing pressure flow (green) and GFW streaming flow (red) are illustrated on the left half within the microfluidic channel

The set-up required for the counter-flow experiment is presented in Fig. 9. The device comprises a base MAWA silicon chip and a PDMS cap bonded to the former, as shown in Fig. 9a–c. The device’s cap is a PDMS microfluidic layer with a 200 µm-wide, 70 µm-high main channel that traces the path of the underlying membrane, such that the microchannel walls enclose the part of the membrane waveguide on the covered half of the device. This main channel is fed by two tributary channels, Fig. 9d, which connect the main channel to the inlet on the far left, where the fluid and particles are pumped into the microfluidic structure. Note that the patterned microfluidic cap encapsulates only the left half of the MAWA chip, while the right half is uncapped. Therefore, the ends of the main channel are not sealed but rather exposed to the ambient. As such, fluid pumped from the inlet on the left flows toward the right and out of the microfluidic device due to the pressure difference and floods the uncapped region of the chip.

**Fig. 10:**
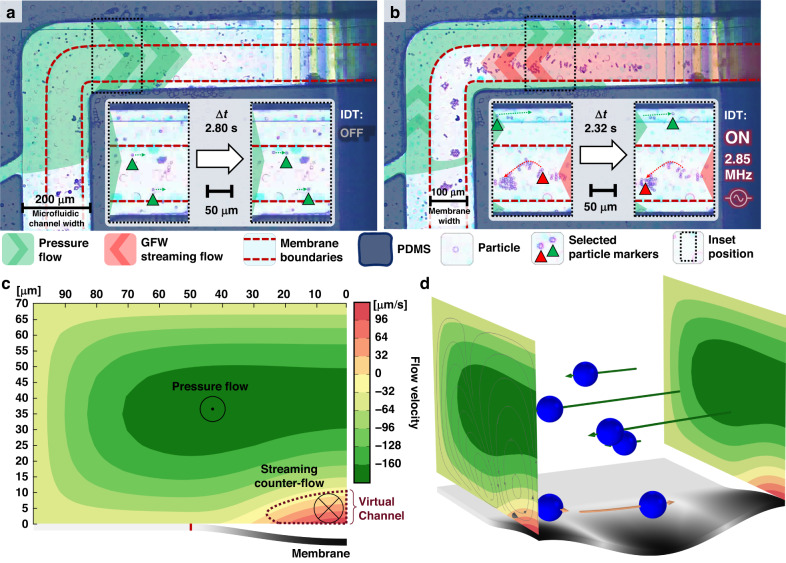
Experimental results of in-channel counter-flow virtual channel generation against a background pressure flow onto the MAWA fitted with a patterned PDMS cap. The background pressure flow spanning the width of the microchannel (200 µm) is indicated in green, while the GFW streaming flow, spanning only the width of the membrane waveguide (100 µm), is displayed in red. **a** Initial state of the systems with IDT off. Particles drift from left to right everywhere across the width of the channel due to the established pressure flow. **b** The IDT is turned on, and GFWs traveling from right to left generate a localized streaming flow against the pressure flow only within the width of the membrane waveguide, as manifested by the particles’ trajectory. The insets in (**a**, **b**) show two still frames spaced in time (Δt) to highlight the trajectory (indicated by the dotted arrows) of selected particles, each identified by a triangle in the color of the type of flow applied to them. **c** Simulated result illustrating the cross-sectional flow inside the channel (half due to symmetry). The GFW-induced streaming gives rise to the counter-flow virtual channel, where particles are transported in the opposite direction from the main pressure flow. **d** 3D simulated results illustrating the presence of both the pressure flow and the streaming-induced counter-flow inside the microchannel acting on blue particles

In the initial state of the experiment, the pressure flow drives the particle-laden fluid inside the microfluidic channel, as shown in Fig. 10a. Under the pressure flow alone, particles circulated inside the microchannel and exited through its lateral outlets to reach the open area, where a sessile droplet was slowly being formed as the fluid exited the channel and no external pressure restriction was in place. The flow velocity profile inside the channel would then correspond to the simulated background pressure flow illustrated in the Fig. 4a. Once the MAWA device was turned on (2.85 MHz at 10 V_pk-pk_), a localized counter-flow was generated on top of the membrane waveguide across both capped and uncapped parts of the device. Inside the channel, the streaming-induced counter-flow is confined within the first 10 µm above the membrane and behaves like a virtual channel by capturing the particles within its limited region and transporting them in the direction of the streaming flow, against the pressure flow. Particles out of reach of the acoustofluidic forces inside the virtual channel were still subjected to the main pressure flow and exited the channel, Fig. 10d. Inside the ambient uncapped region, the GFWs were still active, and particles previously ejected into the open region were recollected onto the membrane, transported toward the channel outlet and seen trickling back inside the PDMS channel, Fig. 10b. The flow velocity profile inside the microfluidic channel during the experiment is represented in the simulation results from Fig. 10c, d, showing both the GFW-induced streaming counter-flow (red) and the main pressure flow (green). A video of the experiment for in-channel localized counter-flow virtual channel generation induced by GFWs is available as the supplementary movie S3.

The in-channel MAWA experiment in Fig. 10 demonstrates localized acoustofluidic manipulation of particles via a GFW-generated counter-flow virtual channel inside a pressure-driven microfluidic channel. The compatibility of the MAWA with standard in-channel microfluidic technologies and the selectivity of this novel acoustofluidic tool powered by GFWs elevate the MAWA as a promising platform for various biomedical applications for particle transport, enrichment, and sample washing.

## Discussion

This study delves into the capability of the MAWA technology to leverage both the ARF and the ASF for advanced localized particle control. In the first part, the acoustofluidic effects of different propagation modes adopted by GFWs, namely the traveling wave, the standing wave, and the rotating wave, were investigated based on 3D mechano-acoustofluidic simulations performed in COMSOL Multiphysics ®.

The simulation results revealed that acoustic streaming, a 2nd-order effect, plays an essential role in flexural wave acoustofluidics by pumping the liquid in the first few micrometers above the actuated membrane waveguide when a traveling wave is present. The pumping observed is dominant in the direction of the traveling wave, while lateral vortical streaming also symmetrically arises due to the finite width of the traveling wave. Particles transported by a traveling wave, whether linearly or circularly, are then aligned along the ridgeline of the traveling anti-nodes formed by the wave train while being pushed forward in the direction of propagation via the ASF. Linearly traveling GFWs hence cause a particle train advancing in the direction of the wave propagation while a rotating wave generates a circular particle train, like a rotating ring trap.

When no traveling component is present inside the wave, such as in the standing wave, the ARF may outperform the lateral ASF and pushes the particles toward the displacement node, forming a static cluster. Nonetheless, the balance between these two forces is not only affected by the actuation mode’s shape but also by other experimental parameters, such as the frequency of actuation and particle size, which will impact the magnitude of both forces differently.

Still, in all cases, the ARF has been observed to push particles downward, keeping them in proximity with the active membrane waveguide bearing the GFWs. Due to the phase velocity of traveling flexural waves being lower than the speed of sound in water, the acoustofluidic fields generated by the waves are evanescent in the medium, leading to highly-localized acoustofluidic effects limited to the first tens of micrometers above the membrane waveguide. Particles out of reach of the forces generated are then left undisturbed inside the liquid sample.

The cavity-agnostic property of flexural-wave-powered acoustofluidic effects enabled by the MAWA allows these distinctive types of localized pumping and trapping to be used effortlessly inside both sessile droplets and standard microfluidic channels. The simulated results of the primary vibration mode are used as mechano-acoustofluidic building blocks to support the second part of the study focused on advanced particle manipulation experiments performed by the MAWA, both inside a sessile droplet and a microfluidic channel. The first in-sessile-droplet experiment showed that 5 and 10 µm particles could be mixed by leveraging the acoustofluidic effects of rotating and static clusters. This way, particles can be brought in contact, mixed, and pushed into clustered formations, a recurrent procedure in several biomedical assays. The second in-sessile-droplet experiment demonstrated particle separation based on their size. A cluster of 5 and 10 µm particles was subjected to both the ARF and the ASF on top of the actuated membrane waveguide. Factors such as the varying intensity of the forces across the membrane and the difference in scaling between the two forces in relation to the particles’ radius led to smaller particles escaping the acoustic trap and gathering further away on the membrane waveguide. This local migration of the small 5 µm particles effectively created two separate groups inside the region of interest in the sessile droplet sample. The third experiment presented occurred inside a microfluidic channel where traveling GFWs generated a streaming counter-flow virtual channel flowing against the established main pressure flow. Particles originally pushed outside the microfluidic channel by the pressure flow could be seen trickling back inside from being transported by the counter-flow virtual channel. This type of manipulation could be used to concentrate particles or refresh the sample solution for practices like cell washing in biomedical research and further consolidate the role of the MAWA as a novel acoustofluidic technology providing advanced manipulation techniques to in-microchannel microfluidics.

Together, the simulations of GFW-based acoustofluidics and the advanced manipulation demonstrations performed inside sessile droplets and microfluidic channels positively assess the ability of the MAWA technology to perform essential particle manipulation tasks inside various microfluidic settings. By leveraging the distinctive mechano-fluidic coupling of flexural waves populating its thin piezoelectric membrane waveguide, the MAWA generates highly-localized evanescent acoustofluidic fields enabling unique on-chip particle control functions. As such, a MAWA chip can dynamically perform fundamental manipulation functions like sample transportation, trapping, patterning, mixing, and separation, which figure at the core of biomedical applications such as cell spheroid and organoid production, local cell and particle enrichment, patterning, and assays. With these advanced capabilities, the advantages resulting from microfabrication for batch fabrication and first-rate reproducibility qualities, and the adaptability granted by its photolithographically defined membrane waveguide, the MAWA presents itself as an innovative cavity-agnostic acoustofluidic platform for the development of future lab-on-a-chip systems.

## Materials and methods

### PDMS cap Fabrication

A PDMS microchannel with a height of 70 μm and a width of 200 μm was fabricated by standard soft lithography using SU-8 2035 (Kayaku) negative photoresist for the master mold. The Sylgard 184 Silicone Elastomer Curing Agent and Base (Dow Corning) were mixed at a 1:10 weight ratio, and then cast on top of the SU-8 mold. Air bubbles were removed by placing the dish inside a vacuum chamber for 1 h. The PDMS was then cured at 50 °C for 3 h. To bond a cut piece of PDMS negative stamp forming the cap with the MAWA chip, both pieces are placed inside an ozone plasma chamber for 3 min and manually aligned and bonded under the microscope.

### Microscopy

The experiments were conducted using a non-inverted reflection microscope (Olympus BX53M) mounted with a video camera (Olympus DP27) to record the acoustofluidic manipulations. The PCB bearing the MAWA chip was connected to a signal generator (Agilent 33220 A) via SMA cables to generate the continuous sinusoidal actuation signal. The signal amplitude reached 6.5–10 V_pk–pk_ for all the IDTs, while the frequencies ranged from 2.5–3.2 MHz for IDT-VN and IDT-VS, and 9.8 MHz for IDT-HE and IDT-HW.

### Sample preparation

The experiments were carried out based on an aqueous solution containing red (5 μm) and blue (10 μm) polystyrene particles (Magsphere, USA). The surfactant Pluronic F127 at 0.5% m/m was also added. For in-droplet experiment, the top of the droplet was flattened with a glass slide supported by conformable sticky bumpers to facilitate the observation under the microscope. The glass slide was hence positioned around 1 to 2 mm above the device, which is far greater than the depth of the evanescent acoustic fields generated by the MAWA.

### Supplementary information

Supplementary Material

Supplementary Movie S1: Particle Mixing

Supplementary Movie S2: Particle Segregation

Supplementary Movie S3: Virtual Channel Counter-flow Generation

## Data Availability

All the data needed to evaluate the conclusions of this work are present in the paper and in the Supplementary Materials.
