# Supplementary material for: Cavity-agnostic acoustofluidic manipulations enabled by guided flexural waves on a membrane acoustic waveguide actuator
Source: Microsyst Nanoeng. 2024 Mar 8;10:33. doi: 10.1038/s41378-023-00643-8 (PMC10920796; doi:10.1038/s41378-023-00643-8)
Supplement: Supplementary file 1 — Supplementary Material [file 41378_2023_643_MOESM1_ESM.docx]

**Supplementary Materials**

Cavity-Agnostic Acoustofluidic Functions Enabled by Guided Flexural Waves on a Membrane Acoustic Waveguide Actuator

Philippe Vachon^1,2*^, Srinivas Merugu^2^, Jaibir Sharma^2^, Amit Lal^2,3^, Eldwin J. Ng^2^, Yul Koh^2^, Joshua E.-Y. Lee^2,4^, Chengkuo Lee^1^

^1^ Department of Electrical and Computer Engineering, National University of Singapore, Singapore.

^2^ Institute of Microelectronics, Agency for Science, Technology and Research (A*STAR), Singapore.

^3^ SonicMEMS Laboratory, School of Electrical and Computer Engineering, Cornell University, Ithaca, USA.

^4^ School of Electrical and Data Engineering, University of Technology Sydney, NSW, Australia.

^*^Correspondence: Philippe Vachon; [Philippe.vachon@u.nus.edu](mailto:Philippe.vachon@u.nus.edu)

This file includes:

Supplementary Text

- COMSOL Multiphysics simulation details

Table S1 Material properties used for numerical simulations

Table S2 Acoustofluidic forces definition in COMSOL

Figures S1 and S2

Legend for movies S1 to S3

References (92-94)

COMSOL Multiphysics simulation details

The acoustofluidic models simulated for this paper rely on several physics and multiphysics interfaces enabled by the software COMSOL Multiphyscis ® (v6.0, Stockholm, Sweden). The simulation steps first go through a Solid Mechanics simulation in the frequency domain to recreate the traveling or standing wave propagating on the membrane waveguide of the MAWA. The solid physics is fully coupled with the acoustic physics from the Pressure Acoustics, Frequency Domain (ACPR) interface inside the frequency domain solver through the Acoustic-Structure Boundary Multiphysics interface applied to all the boundaries acting as an interface between the solid and the fluid domain. This allows for the mechanical displacement generated on the free part of the solid domain (see domains in-between the green and the red lines in Figure S1 and Figure S2) to become an acoustic source in the fluid. External surfaces that are not covered by the Acoustic-Structure Boundary multiphysics are set to different boundary conditions depending on the model studied. For the channel model, a Thermoviscous Boundary Layer Impendence with a no slip mechanical condition is assigned to the channel walls (in blue in Figure S1). The end boundaries however are set to Periodic Condition with a continuity periodicity to mimic an infinitely long channel. For the in-droplet hemispherical model, a spherical Perfectly Matched Layer (PML) artificial domain is used to simulate a very large fluidic domain. The length of the PML is set to match one full acoustic wavelength in water, around 0.5 mm. In the Pressure Acoustic interface, the pressure dependent variable has its discretization set to Cubic Lagrange to increase the uniformity of the mesh refinement. The values used for the anisotropic Linear Elastic Material (phosphorus-doped Si), the stress-charge form Piezoelectric Materials (AlScN) and the water are present in Table S1.

Once the Solid Mechanics and the Pressure Acoustics are solved by the Frequency Domain step (Suggested direct solver, MUMPS), the following step is a stationary step to model the acoustic streaming occurring as a second-order effect from the first-order pressure and velocity perturbations using a Laminar Flow (SPF) fluid interface. The fluid interface and the acoustic fields modelled by the Pressure Acoustics are coupled via two coupling Multiphysics, the Acoustic Streaming Domain Coupling and the Acoustic Streaming to compute the forces, stresses and boundary slip velocities inside the bulk fluid and at the acoustic source boundaries. All the solid boundaries or the hard walls are added as a boundary contribution to the multiphysics while all the fluidic domains, expect the PML domains if present, are used for the domain coupling. In the hemispherical model, a normal stress $f_{0}=0$ N/m² Open Boundary condition is placed at the interface between the PML and the fluidic domain. For the in-channel model, the remaining opposing ends are set to a Periodic Flow Condition of $\Delta p = 0$ Pa to indicate there is no driving pressure flow at the second-order. A Pressure Point Constraint $p_{0} = 0$ Pa is set on a bordering point to facilitate the convergence of the fully-coupled Stationary Solver (Direct, fluid flow variables, PARDISO).

For in-channel simulation, an extra Laminar Flow physics interface is added to independently simulate the background pressure flow caused by the difference in pressure between the two ends of the channel. A Periodic Flow Condition is introduced with $\Delta p = [0, 12, 24, 36]$ Pa as a variable to simulate the interaction between the intensity of the background flow and the shape of the counter-flow virtual channel.

The acoustofluidic forces are calculated as manually-defined variables, external to any physics interface, using the dependent variables solved with the ACPR(p1) and SPF(p2,u2,v2,w2) interfaces and additional parameters to compute their xyz-components. For the ASF, the Lagrangian velocity is used as the 2^nd^-order velocity of the fluid around the particle. All manually-defined equations and variables are available in Table S2 Acoustofluidic forces definition in COMSOL.

Please refer to COMSOL’s Acoustics Module User's Guide [92] for further information regarding the ACPR and SPF modules integration.

Table S1 Material properties used for numerical simulations

| **Al_85_Sc_15_N** |  |  |  |  |  |
| --- | --- | --- | --- | --- | --- |
| Density | $\rho_{m}$ |  | 3270 | kg/m³ | Interpolated  from [93] |
| Elasticity matrix | $c_{E}$ |  | $\left[ \begin{matrix} 351 & 143 & 109 & 0 & 0 & 0 \\ 143 & 351 & 109 & 0 & 0 & 0 \\ 109 & 109 & 300 & 0 & 0 & 0 \\ 0 & 0 & 0 & 111 & 0 & 0 \\ 0 & 0 & 0 & 0 & 111 & 0 \\ 0 & 0 & 0 & 0 & 0 & 104 \end{matrix} \right]$ | GPa | Interpolated  from [93], [94] |
| Coupling matrix | $e_{\mathrm{ES}}$ |  | $\left[ \begin{matrix} 0 & 0 & -0.54 & 0 & -0.26 & 0 \\ 0 & 0 & -0.54 & -0.26 & 0 & 0 \\ -0.54 & -0.54 & 1.92 & 0 & 0 & 0 \\ 0 & -0.26 & 0 & 0 & 0 & 0 \\ -0.26 & 0 & 0 & 0 & 0 & 0 \\ 0 & 0 & 0 & 0 & 0 & 0 \end{matrix} \right]$ | C/m² | Interpolated  from [93] |
| Relative permittivity | ε_r_ |  | 12.3 |  |  |
| **Phosphorus-doped Si** | | |  |  |  |
| Density | $\rho_{m}$ |  | 2330 | kg/m³ |  |
| Elasticity matrix | D |  | $\left[ \begin{matrix} 146 & 60 & 60 & 0 & 0 & 0 \\ 60 & 146 & 60 & 0 & 0 & 0 \\ 60 & 60 & 146 & 0 & 0 & 0 \\ 0 & 0 & 0 & 69 & 0 & 0 \\ 0 & 0 & 0 & 0 & 69 & 0 \\ 0 & 0 & 0 & 0 & 0 & 69 \end{matrix} \right]$ | GPa |  |
| **Water** (25 °C) |  |  |  |  |  |
| Density | $\rho_{w}$ | acpr.rho | 997.03 | kg/m³ |  |
| Speed of sound | c | acpr.c | 1495.3 | m/s |  |
| Dynamic viscosity | $\mu$ | eta0 | 0.89255 | mPa s |  |
| Bulk viscosity | $\mu_{B}$ |  | 2.4902 | mPa s |  |
| **Polystyrene Particles** | | |  |  |  |
| Diameter | d | dpo | 5-10 | µm |  |
| Density | $\rho_{p}$ | rhopo | 1050 | kg/m³ |  |
| Pressure wave speed | $c_{p-p}$ | cp_p | 2350 | m/s |  |
| Shear wave speed | $c_{s-p}$ | cs_p | 1150 | m/s |  |
| Compressibility | $\kappa_{p}$ | kappasp | $\left[ \left( c_{p-p}^{2}-{\frac{4}{3}c}_{s-p}^{2} \right)\rho_{p} \right]^{-1}$ | 1/Pa |  |

Table S2 Acoustofluidic forces definition in COMSOL

| Vector Transform: 1st-Order Velocity | |  |
| --- | --- | --- |
| v1_ac 1^st^-Order Velocity Vector Field | | m/s |
| acpr.vx | acpr.vy | acpr.vz |

| Vector Transform: 2nd-Order Velocity | |  |
| --- | --- | --- |
| v2_ac 2nd-Order Lagrangian Velocity Vector Field | | m/s |
| vx_LG_acpr | vy_LG_acpr | vz_LG_acpr |

| Lagrangian Velocity Variables | |  |  |
| --- | --- | --- | --- |
| vx_SL_acpr | 0.5*real((d(conj(acpr.v_tx),x)*acpr.v_tx+d(conj(acpr.v_tx),y)*acpr.v_ty+d(conj(acpr.v_tx),z)*acpr.v_tz)/acpr.iomega) | m/s | Stokes slip, x-component |
| vy_SL_acpr | 0.5*real((d(conj(acpr.v_ty),x)*acpr.v_tx+d(conj(acpr.v_ty),y)*acpr.v_ty+d(conj(acpr.v_ty),z)*acpr.v_tz)/acpr.iomega) | m/s | Stokes slip, y-component |
| vz_SL_acpr | 0.5*real((d(conj(acpr.v_tz),x)*acpr.v_tx+d(conj(acpr.v_tz),y)*acpr.v_ty+d(conj(acpr.v_tz),z)*acpr.v_tz)/acpr.iomega) | m/s | Stokes slip, z-component |
| vx_LG_acpr | u2+vx_SL_acpr | m/s | Lagrangian velocity, x-component |
| vy_LG_acpr | v2+vy_SL_acpr | m/s | Lagrangian velocity, y-component |
| vz_LG_acpr | w2+vz_SL_acpr | m/s | Lagrangian velocity, z-component |

| Acoustic Streaming Force Variables | |  |  |
| --- | --- | --- | --- |
| ASFx_ac | mp*(nojac(v2_ac.u1))/taup | N | ASF, x-component |
| ASFy_ac | mp*(nojac(v2_ac.u2))/taup | N | ASF, y-component |
| ASFz_ac | mp*(nojac(v2_ac.u3))/taup | N | ASF, z-component |
| ASF_ac | sqrt(ASFx_ac^2+ASFy_ac^2+ASFz_ac^2) | N | ASF |
| taup | rhopo*dpo^2/(18*nojac(eta0)) | s | Particle velocity response time |
| mp | rhopo*pi*dpo^3/6 | kg | Particle mass |

| Acoustic Radiation Force Variables | |  |  |
| --- | --- | --- | --- |
| ARFx_ac | −2*apo^3*pi*(kappas1_ac*real(conj(f0sl_ac)*conj(nojac(p1_ac))*nojac(d(p1_ac,x)))/3−0.5*rho1_ac*real(conj(f1sl_ac)*(nojac(d(v1_ac.u1,x))*conj(nojac(v1_ac.u1))+nojac(d(v1_ac.u1,y))*conj(nojac(v1_ac.u2))+nojac(d(v1_ac.u1,z))*conj(nojac(v1_ac.u3))))) | N | Acoustofluidic radiation force, x-component |
| ARFy_ac | −2*apo^3*pi*(kappas1_ac*real(conj(f0sl_ac)*conj(nojac(p1_ac))*nojac(d(p1_ac,y)))/3−0.5*rho1_ac*real(conj(f1sl_ac)*(nojac(d(v1_ac.u2,x))*conj(nojac(v1_ac.u1))+nojac(d(v1_ac.u2,y))*conj(nojac(v1_ac.u2))+nojac(d(v1_ac.u2,z))*conj(nojac(v1_ac.u3))))) | N | Acoustofluidic radiation force, y-component |
| ARFz_ac | −2*apo^3*pi*(kappas1_ac*real(conj(f0sl_ac)*conj(nojac(p1_ac))*nojac(d(p1_ac,z)))/3−0.5*rho1_ac*real(conj(f1sl_ac)*(nojac(d(v1_ac.u3,x))*conj(nojac(v1_ac.u1))+nojac(d(v1_ac.u3,y))*conj(nojac(v1_ac.u2))+nojac(d(v1_ac.u3,z))*conj(nojac(v1_ac.u3))))) | N | Acoustofluidic radiation force, z-component |
| kappas1_ac | 1/(c1_ac^2*rho1_ac) | 1/Pa | Compressibility (isentropic) |
| kappasp | 1/((cp_p^2-4*cs_p^2/3)*rhopo) | 1/Pa | Particle Compressibility (isentropic) |
| f0sl_ac | 1−kappasp/kappas1_ac |  | Particle monopole scattering coefficient |
| f1sl_ac | 2*(−1+rhopo/rho1_ac)/(1+2*rhopo/rho1_ac) |  | Particle dipole scattering coefficient |
| ARF_ac | sqrt(ARFx_ac^2+ARFy_ac^2+ARFz_ac^2) | N | Acoustofluidic radiation force |
| rho1_ac | acpr.rho | kg/m³ | Fluid density |
| c1_ac | acpr.c | m/s | Speed of sound in fluid |
| p1_ac | p1 | Pa | 1^st^-order pressure variable solved in ACPR. |

**
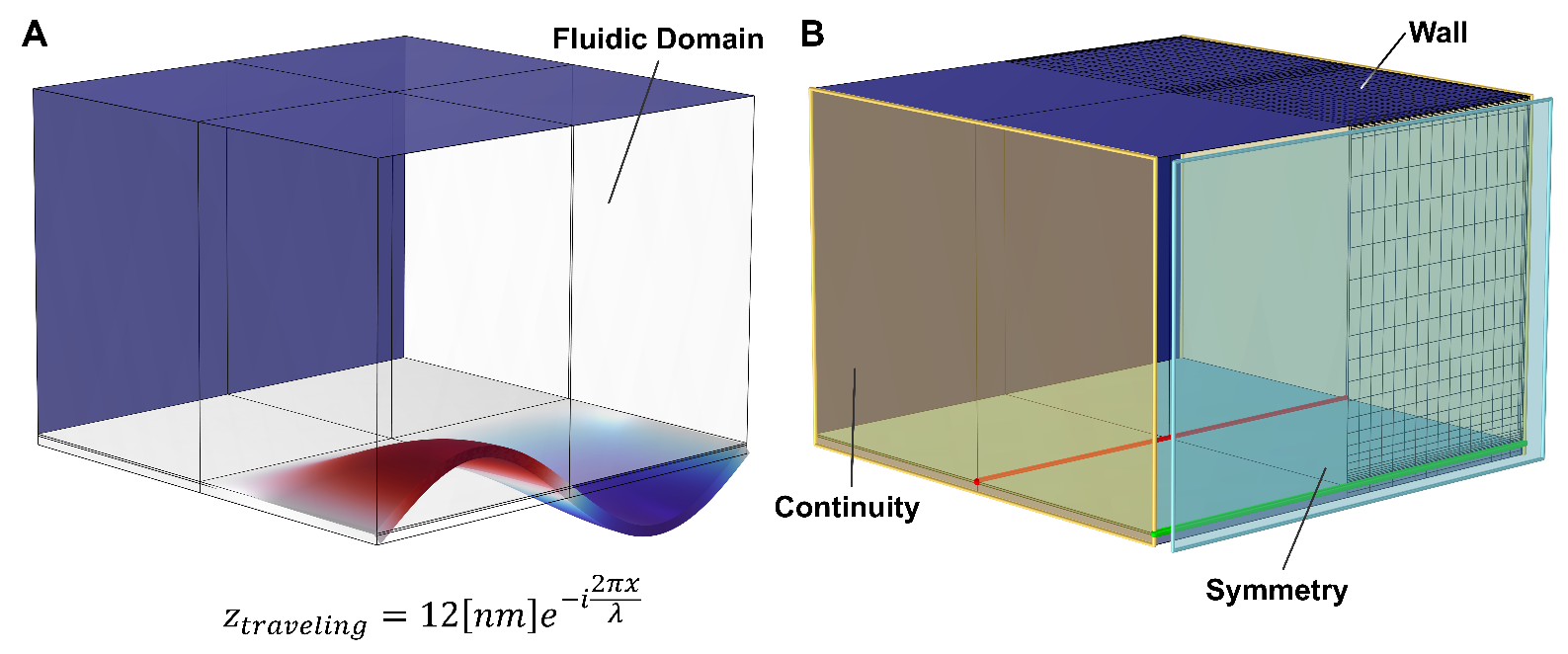
**

Figure S1 Rectangular domain used for the in-channel acoustofluidic simulations of rectilinearly traveling guided flexural waves. (A) Opaque solid domain with displacement color mapping showing a full wavelength of a flexural wave. The transparent domain above the solid domain forms the liquid domain bounded by the channel’s solid walls (blue). (B) Rectangular model with colored parts and surfaces showing the different regions and the surfaces with special boundaries. The dark blue surfaces are the solid walls bounding the channel. The framed cyan translucent plane is the symmetry plane reducing the fluidic domain by half. The framed yellow translucent surfaces are the continuity boundaries for the mechanical motion, and the acoustic fluidics variables like the pressure and the velocity. The green line is the actuation boundary for the traveling flexural wave in the same plane as the symmetry plane. It follows the constrained displacement function $z_{\mathrm{traveling}}$ to generate the traveling flexural wave. The solid region between the green and the red lines forms the pSON membrane waveguide, while the region beyond the red line is fixed.


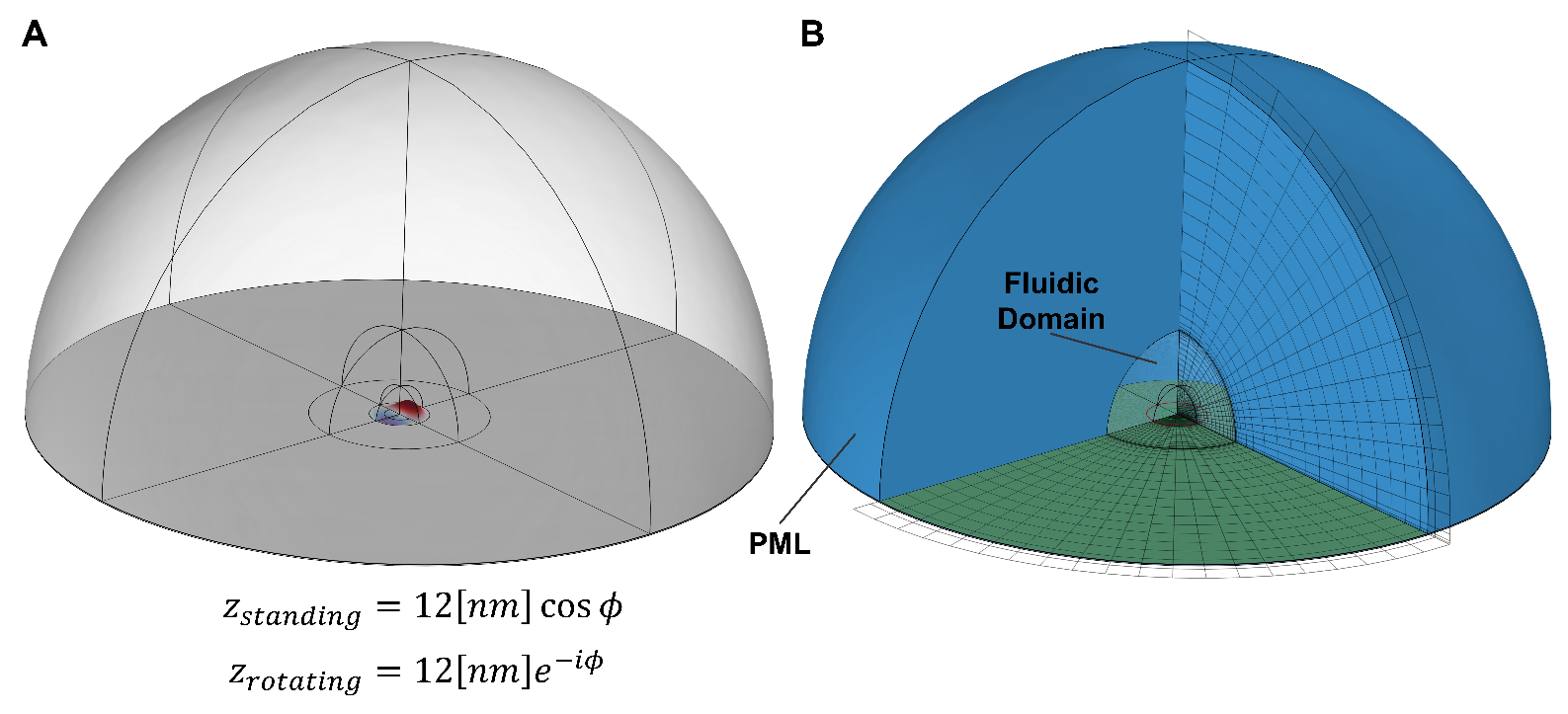


Figure S2 Hemispherical domain used for in-droplet acoustofluidic simulations of rotating waves and standing flexural waves. (A) Opaque solid domain with displacement color mapping showing two displacement anti-nodes around a node. The transparent domain above the solid domain is the liquid domain forming a part of the droplet. (B) Hemispherical model with colored parts showing the different regions. The blue region is the PML layer surrounding the fluidic domain in the center. The green plate is the top of the silicon chip covered with a thin piezoelectric film. Any solid domain inside the red line forms the pSON membrane and is free to move while the rest is fixed. The green line is the actuation boundary for the rotating and standing waves. It follows the constrained displacement function $z_{\mathrm{standing}}$ or $z_{\mathrm{rotating}}$, where $\phi$ is the azimuthal angle in the plane of the bottom surface in cylindrical coordinates.

Legend for movies S1 to S3

Movie S1. Acoustofluidic experiment of particle mixing. Two groups of particles of different sizes are continuously mixed by the acoustofluidic forces on top of the actuated membrane waveguide. The membrane waveguide is actuated at 2.85 MHz by the IDT-VN and IDT-VS, forming vortical traps on the lateral segments of the MAWA device. After a short period, the actuation frequency is changed to 2.77 MHz, which changes the actuation mode. The mixed particles are now trapped in static clusters forming a linear array on the membrane waveguide segment.

Movie S2. Acoustofluidic experiment of spatial segregation of particles based on their sizes. At the start of the experiment, the particles are gathered on the membrane waveguide in a single cluster near IDT-VN. Shortly after, IDT-HE and IDT-HW are actuated (9.8 MHz, 10 V_pk-pk_) which sends counter-propagating flexural waves toward the top membrane segment and forms a field of standing waves with trapping properties. Then, IDT-VN is actuated (3.19 MHz, 10 V_pk-pk_), continuously generating traveling guided flexural waves inducing a localized streaming pushing the group of particles clockwise along the membrane waveguide. As the particles migrate from one trap to another, smaller particles are seen to be slowly escaping the bulk of the cluster. In the second part of the movie, the voltage amplitude at IDT-Hs is progressively turned down to 6.5 V. This voltage shift causes the effective trapping of the acoustofluidic effects to weaken, leading to the smaller particles escaping the traps and gathering further down the vertical segment, effectively creating a spatial segregation between the original mixed group and the new group almost exclusively formed of small 5 µm red particles.

Movie S3. Acoustofluidic experiment of localized counter-flow virtual channel generation inside a microfluidic channel. A background pressure flow is present throughout the experiment and carries particles across the channel from left to right. A moment later, IDT-VN (3.2 MHz, 10 V_pk-pk_) is actuated, and a traveling wave propagates along the membrane waveguide, generating a localized streaming flow able to capture, align and transport particles in the direction of wave propagation. The streaming flow is positioned against the main pressure flow, forming a virtual channel transporting particles in the opposite direction. Particles initially ejected out of the channel on the right-side trickle back inside the channel and flow from right to left inside the streaming-induced virtual channel.

References

[92] “Acoustics Module User’s Guide,” *COMSOL Multiphysics® v. 6.1*. COMSOL AB, Stockholm, Sweden, 2022, [Online]. Available: https://doc.comsol.com/6.1/doc/com.comsol.help.aco/AcousticsModuleUsersGuide.pdf.

[93] N. Kurz *et al.*, “Experimental determination of the electro-acoustic properties of thin film AlScN using surface acoustic wave resonators,” *J. Appl. Phys.*, vol. 126, no. 7, p. 075106, Aug. 2019, doi: 10.1063/1.5094611.

[94] M. A. Caro *et al.*, “Piezoelectric coefficients and spontaneous polarization of ScAlN,” *J. Phys. Condens. Matter*, vol. 27, no. 24, p. 245901, Jun. 2015, doi: 10.1088/0953-8984/27/24/245901.
